# Impact of left ventricular trabeculations and papillary muscles on measures of cavity volume and ejection fraction

**DOI:** 10.1186/1532-429X-13-S1-P36

**Published:** 2011-02-02

**Authors:** Michael L Chuang, Philimon Gona, Gilion LTF Hautvast, Carol J Salton, Susan J Blease, Susan B Yeon, Marcel Breeuwer, Christopher J O'Donnell, Warren J Manning

**Affiliations:** 1 The NHLBI's Framingham Heart Study, Framingham, MA, USA; 2 Philips Healthcare, Best, Netherlands; 3 Beth Israel Deaconess Medical Center, Boston, MA, USA

## Introduction

Left ventricular (LV) trabeculations are typically considered LV cavity volume (i.e. ignored) when analyzing cardiac magnetic resonance (CMR) images as they are difficult to manually segment, but they are not actually part of the LV bloodpool. The treatment of papillary muscles is more variable, but they too have often been considered bloodpool.

## Purpose

To assess the impact of LV trabeculations and papillary muscles on LV volumes and ejection fraction (EF) determined from CMR images using automated segmentation software.

## Methods

Contiguous 10-mm thick cine CMR images encompassing the left ventricle in the short-axis orientation (SSFP: TR/TE/FA=3.2ms/1.6ms/60°, 208x256 matrix, 400-mm FOV; 1.5-T scanner, Philips Healthcare, Best, the Netherlands) from 1494 adults (aged 64±9 yrs, 795 women) in the Framingham Heart Study Offspring cohort were analyzed using semi-automated epi- and endocardial LV border detection to determine LV end-diastolic and end-systolic volumes (EDV, ESV), EF and LV mass (LVM) before and after “adjustment” (ADJ) for trabeculations and papillary muscles. ADJ was fully automated and used fuzzy grayscale thresholding capable of accounting for partial volume effects (Cardiac Explorer, Philips Healthcare). Two-sample t-test was used to assess between-sex differences and paired t-test was used to evaluate effect of ADJ. Intra-class correlation (ICC) was used to assess reproducibility on 48 randomly selected participants from equal strata of sex and age-tertile.

## Results

Prior to ADJ, men had greater EDV, ESV and LVM than women, while women had higher EF (Table 1, mean±standard deviation). ADJ revealed that trabeculations and papillary muscles occupied 22±3 % of LV cavity volume at ED; this proportion was similar for men and women (p=0.64). Total LV trabecular and papillary muscle volume increased with greater LVM (r=0.49, p<0.0001). Post-ADJ LV volumes decreased significantly within each sex (p<0.0001, all), but remained greater in men. Post-ADJ LVEF was greater than pre-ADJ EF in both sexes, and post-ADJ EF remained greater in women than men (p<0.0001). Post-ADJ and pre-ADJ values were highly correlated for EDV, ESV, LVM and EF (r ≥0.95, p<0.0001 for all). ADJ preserved high interobserver reproducibility with Post-ADJ EDV: ICC=0.99, Post-ADJ ESV: ICC=0.99, Post-ADJ LVM: ICC=0.99), Post-ADJ EF: ICC=0.96.

**Table 1:** 

				
Pre-ADJ	EDV (ml)	ESV(ml)	LVM (g)	LVEF

Men	151±32	45±17	106±26	0.71±0.07

Women	112±21	30±10	64±16	0.74±0.06

M vs. W	p<0.0001	p<0.0001	p<0.0001	p<0.0001

				

Post-ADJ	EDV (ml)	ESV (ml)	LVM (g)	LVEF

Men	119±26	25±12	142±30	0.79±0.07

Women	88±17	16±7	91±19	0.82±0.06

M vs. W	p<0.0001	p<0.0001	p<0.0001	p<0.0001

## Conclusions

LV trabeculations and papillary muscles occupy 22% of the putative LV blood pool in both sexes. Adjustment for trabecular and papillary muscle volume results in increased LV ejection fraction. Automated segmentation of LV trabeculations and papillary muscles is highly reproducible.

